# Supplementary material for: An ancient genome of Streptococcus pyogenes from a pre-Columbian Bolivian mummy
Source: Nat Commun. 2026 Apr 13;17:4516. doi: 10.1038/s41467-026-71603-9 (PMC13190766; doi:10.1038/s41467-026-71603-9)
Supplement: Supplementary file 1 — Supplementary Information [file 41467_2026_71603_MOESM1_ESM.pdf]

Supplementary Figures

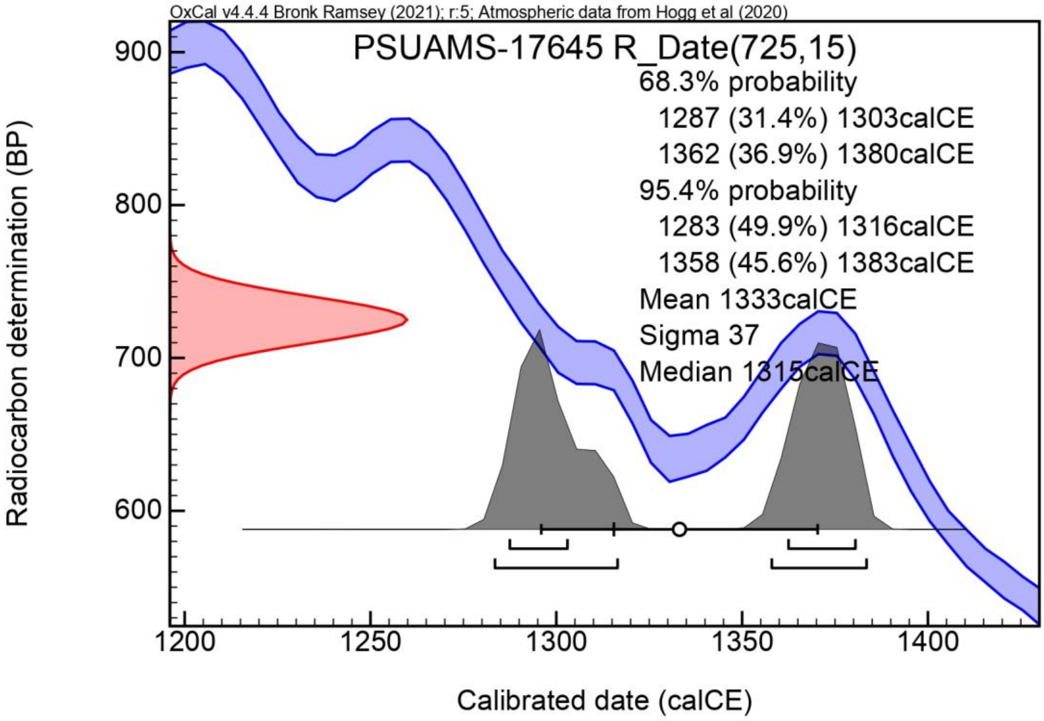

Figure S1: Radiocarbon dates calibrated distribution. Radiocarbon dates were obtained by using the Oxcal computer program (v4.4.4) [1] applying the SHCal20, for the Southern Hemisphere Calibration Curve [2].

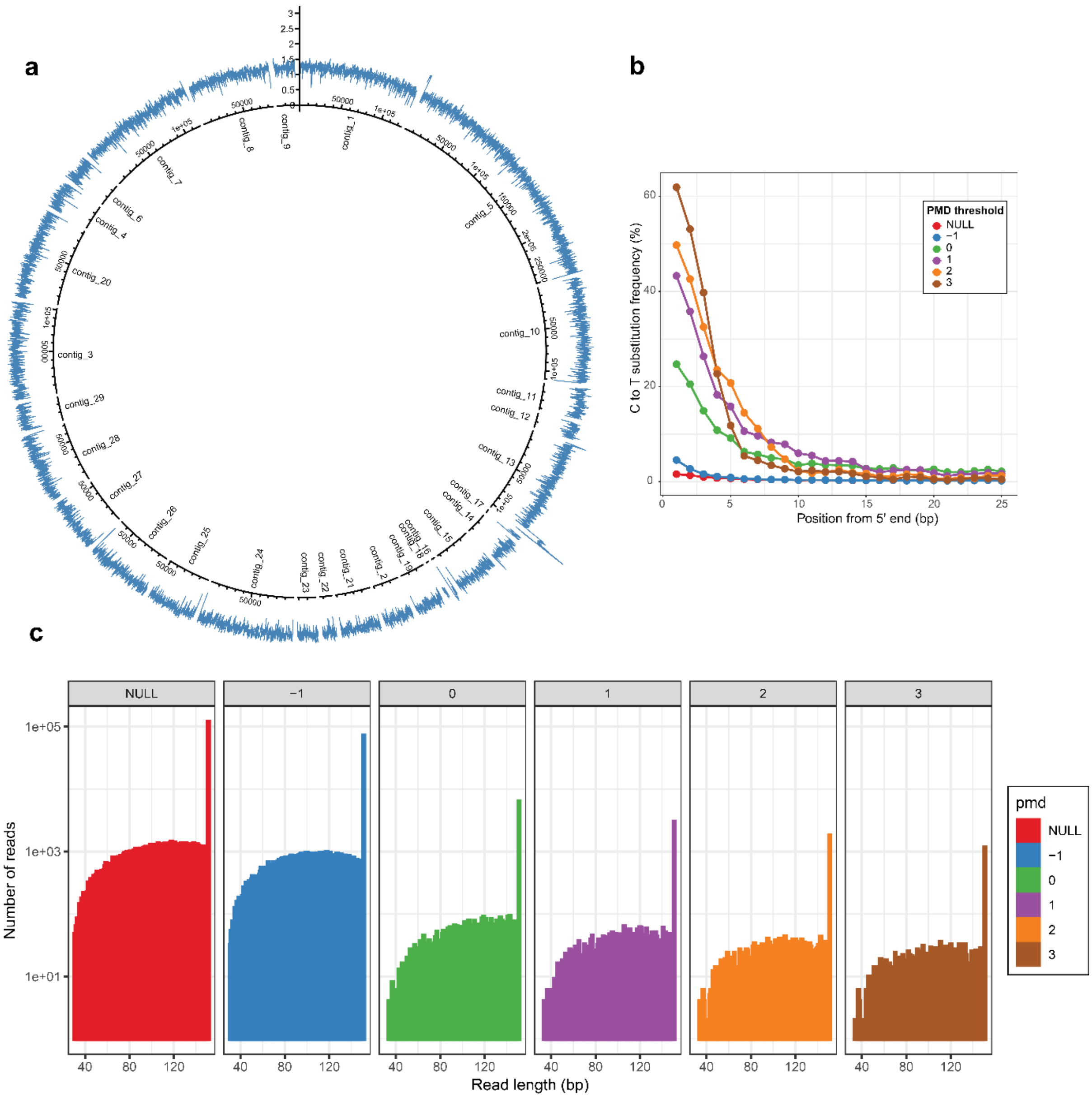

Figure S2. Genome-wide coverage distribution and ancient DNA damage patterns across different PMD tools filtering thresholds. (**a**) Circular plot showing the sequencing coverage depth across all 29 contigs of the assembled genome. Coverage is displayed on a logarithmic scale, in 100 bp bins. Each sector represents an individual contig, with contig names indicated. (**b**) C to T substitution frequency at the 5' end of sequenced reads under different PMD (post-mortem damage) filtering thresholds [3]. (**c**) Read length distributions across different PMD filtering thresholds displayed as faceted plots with logarithmic y-axes. Each panel represents a different PMD threshold (NULL, -1, 0, 1, 2, 3).

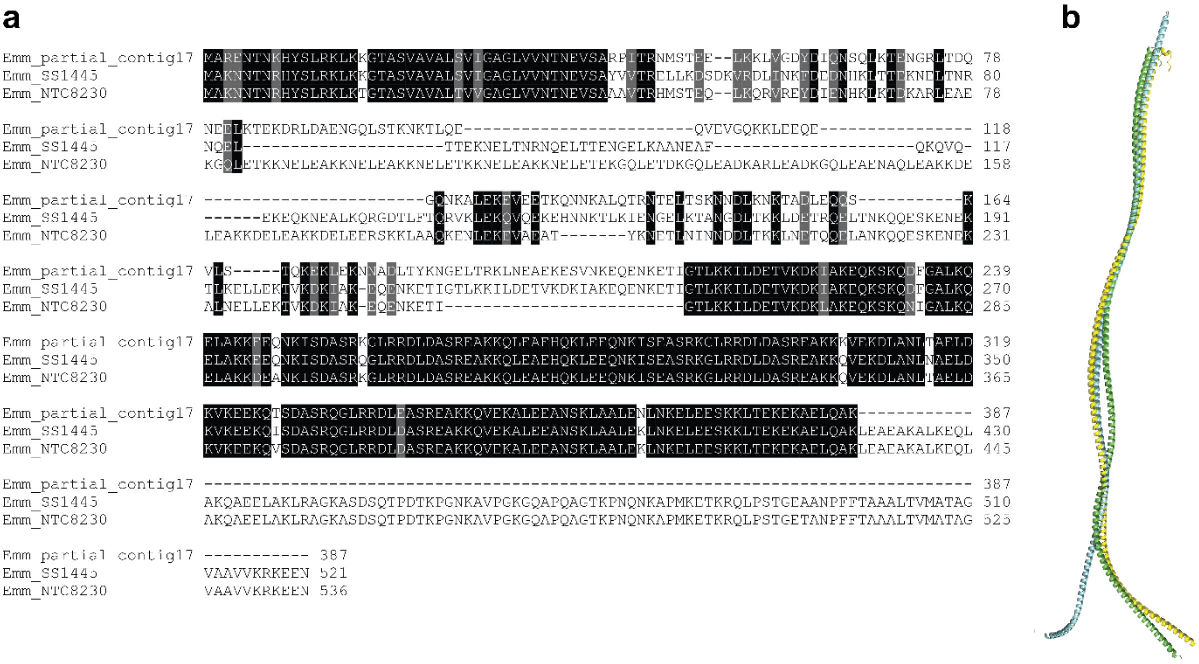

Figure S3. Comparative analysis of the M protein (Emm) reveals unique sequence features in the ancient Bolivian *Streptococcus pyogenes* strain. (**a**) Multiple sequence alignment of M protein (Emm) from the ancient Bolivian strain compared to reference M protein sequences from modern *S. pyogenes* strains. Three representative sequences are shown: Emm_partial_contig17 (partial emm gene from the ancient Bolivian strain), Emm_SS1445 and Emm_NTC8230 (modern reference strains). Black shading indicates identical residues across all sequences, while gray shading shows conserved substitutions. Dashes represent gaps introduced for optimal alignment. (**b**) Three-dimensional structural prediction of the M protein coiled-coil domain. The structure shows the characteristic α-helical coiled-coil conformation of the M protein, rendered as a ribbon diagram. The ancient Bolivian strain (green) is overlaid with reference sequences Emm_NTC8230 (blue) and Emm_SS1445 (yellow) to visualize structural conservation and variation.

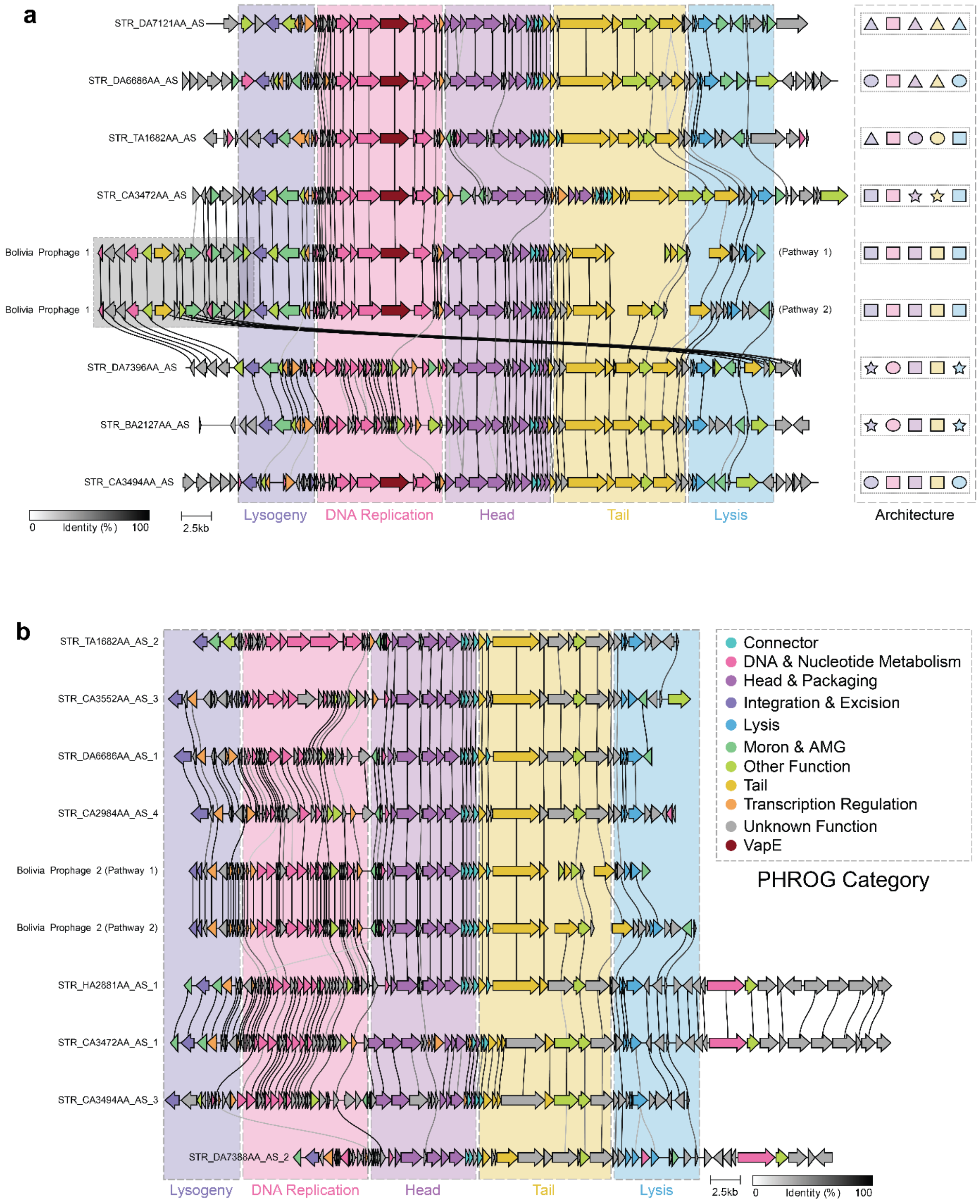

Figure S4. Comparative genomic architecture of prophages from the ancient Bolivian *Streptococcus pyogenes* compared with modern streptococcal prophages. (a) Mosaic architecture of Prophage 1 from the ancient Bolivian *S. pyogenes* strain compared with closely related prophages from modern *Streptococcus* strains. The ancient Bolivian Prophage 1 is shown in two alternative assembly pathways (Pathway 1 and Pathway 2) representing different possible genomic configurations due to assembly ambiguity in the fragmented ancient DNA. Reference prophages from modern strains include

STR_DA7121AA_AS, STR_DA6868AA_AS, STR_TA1682AA_AS, STR_CA3472AA_AS, STR_DA7396AA_AS, STR_BA2127AA_AS, and STR_CA3484AA_AS. Each arrow represents a predicted coding sequence (CDS), with arrow direction indicating gene orientation. (**b**) Mosaic architecture of Prophage 2 from the ancient Bolivian S. pyogenes strain compared with related prophages. Reference sequences include STR_TA1682AA_AS_2, STR_CA3522AA_AS_3, STR_DA6868AA_AS_1, STR_CA2984AA_AS_4, STR_HA2881AA_AS_1, STR_CA3472AA_AS_1, STR_CA3464AA_AS_3, and STR_DA7388AA_AS_2. The ancient Bolivian Prophage 2 is shown in two alternative pathway reconstructions representing different possible genomic organizations.

The IDs of the modern strains refer to the assembly IDs in the EnteroBase database (https://enterobase.warwick.ac.uk/).

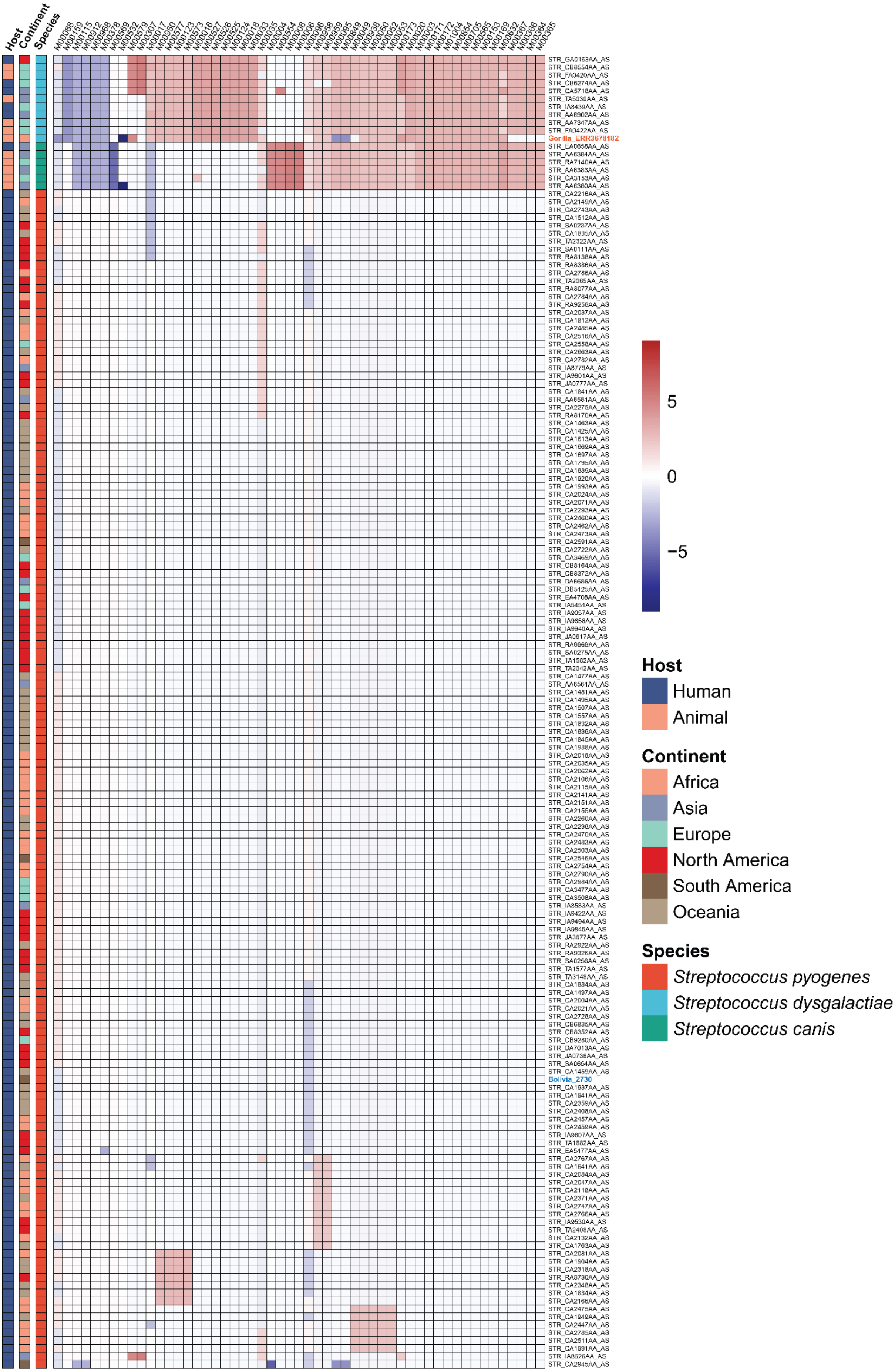
Host
Continent
Species
Gorilla_ERR3678182
Bolivia_2730
5
0
−5
Host
Human
Animal
Continent
Africa
Asia
Europe
North America
South America
Oceania
Species
Streptococcus pyogenes
Streptococcus dysgalactiae
Streptococcus canis

Figure S5. Heatmap displays the counts of enzymes per each KEGG modules, that are significantly differential across samples. Samples are annotated by the continent of origin (Africa, Asia, Europe, North America, South America, Oceania), host type (Human, Animal), and species classification. Module abundance is shown as z-scores. Modules with significant differential abundance between species based on Kruskal-Wallis test (FDR < 0.001) are shown (For full list, please refer to Supplementary Data 16). Bolivia 2730 and Gorilla ERR3678182 samples are highlighted.

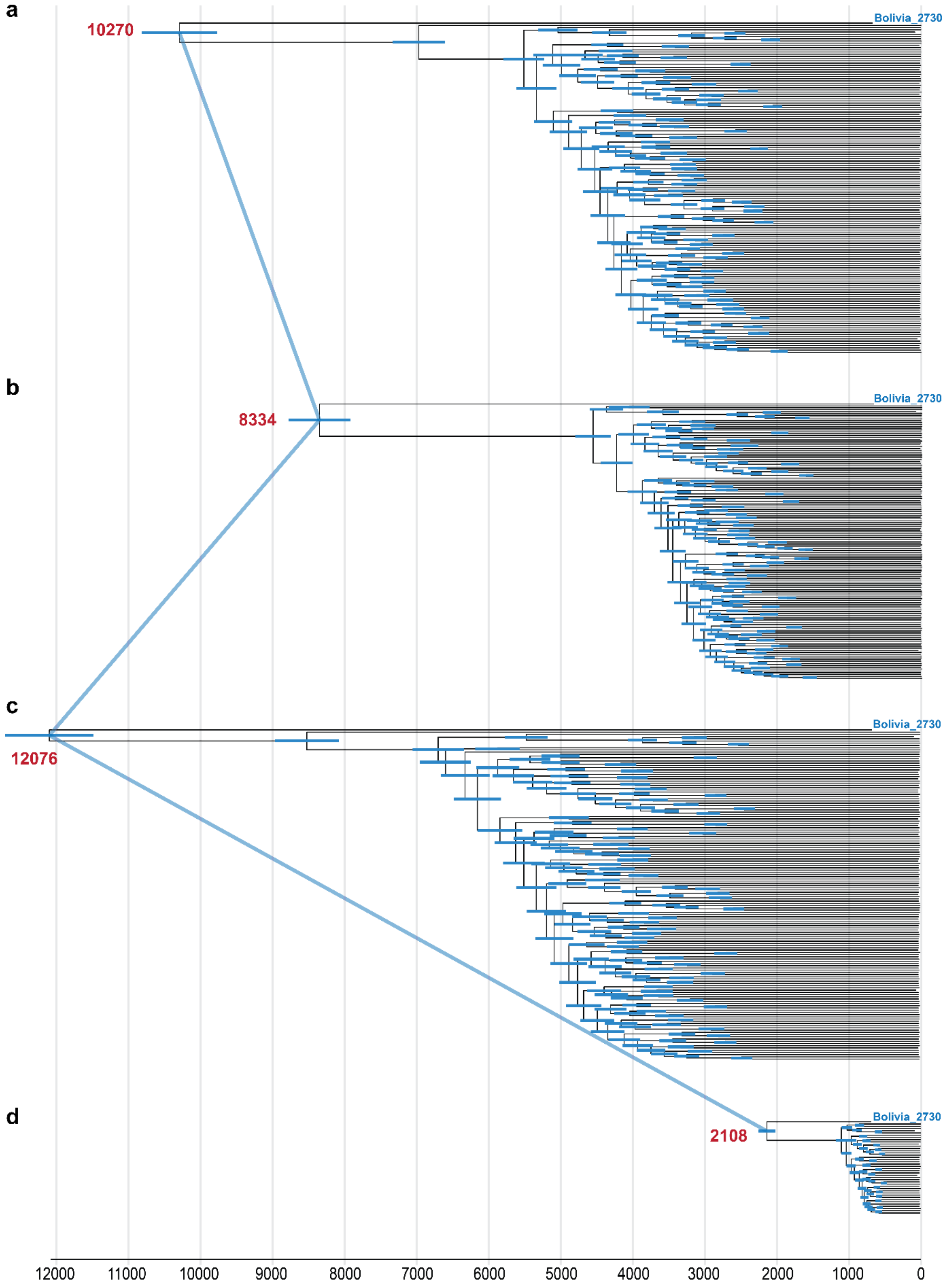

Figure S6. Temporal calibration and phylogenetic placement of the ancient Bolivian Streptococcus pyogenes strain under different evolutionary models and taxon sampling strategies. Time-calibrated phylogenies showing the evolutionary placement of the ancient Bolivian *S. pyogenes* strain (Bolivia_2730, highlighted in blue) relative to

modern strains under different molecular clock models, substitution models, and taxon sampling schemes. Blue bars on nodes represent 95% highest posterior density (HPD) intervals for divergence time estimates. Numbers in red indicate the estimated age (in years before present) of the most recent common ancestor of the clade containing Bolivia_2730 and modern S. pyogenes strains. Time scale is shown on the x-axis in years before present (12,000 to 0 years BP). (**a**) GTR+Γ substitution model with constant coalescent population prior. (**b**) GTR+Γ substitution model with Bayesian Skyline coalescent prior. (**c**) *S. pyogenes*-only dataset with GTR+Γ and constant coalescent prior. To test whether the inclusion of outgroup taxa (*S. dysgalactiae* and *S. canis*) affects the temporal calibration, this analysis includes only *S. pyogenes* strains. (**d**) Reduced taxon set (50 samples) with GTR+Γ and constant coalescent prior.

Supplementary References

1 Bronk Ramsey, C. J. U. h. c. a. o. a. u. o. h. OxCal v. 4.4. 4 [software]. (2021).

2 Hogg, A. G. *et al.* SHCal20 Southern Hemisphere Calibration, 0–55,000 Years cal BP. *Radiocarbon* **62**, 759-778, doi:10.1017/RDC.2020.59 (2020).

3 Skoglund, P. *et al.* Separating endogenous ancient DNA from modern day contamination in a Siberian Neandertal. **111**, 2229-2234, doi:doi:10.1073/pnas.1318934111 (2014).
