# Supplementary material for: An ancient genome of Streptococcus pyogenes from a pre-Columbian Bolivian mummy
Source: Nat Commun. 2026 Apr 13;17:4516. doi: 10.1038/s41467-026-71603-9 (PMC13190766; doi:10.1038/s41467-026-71603-9)
Supplement: Supplementary file 2 — Description of Additional Supplementary Files [file 41467_2026_71603_MOESM2_ESM.pdf]

Description of Additional Supplementary Files:

Supplementary Data 1: Radiocarbon dates and stable isotope data

Supplementary Data 2: Summary stats of shotgun sequencing

Supplementary Data 3: MetaPhlan report

Supplementary Data 4: Comparative data - AMDir Table

Supplementary Data 5: De-novo metagenomic assembly statistics and GTDB taxonomy of bacterial genomes assembled by MEGAHIT

Supplementary Data 6: MLST typing

Supplementary Data 7: Emm typing

Supplementary Data 8: List of *Streptococcus pyogenes* strain (adapted from Frost et al. 2020) that share the same configuration of the Mga regulon with the Bolivian strain.

Supplementary Data 9: List of modern *Streptococcus pyogenes* strains that display highest sequence identity (on DNA and amino acid level) to the partial Bolivian emm gene.

Supplementary Data 10: BLASTp results of the important virulence genes in the ancient Bolivian *S. pyogenes* genome

Supplementary Data 11: Virulence genes as detected with abricate and vfdb database

Supplementary Data 12: Antibiotic resistance genes as detected with abricate and CARD database

Supplementary Data 13: List of *Streptococcus pyogenes* two-component system regulators (adapted from Buckley et al. 2018) and their presence/absence in the Bolivian strain.

Supplementary Data 14: EnteroBase representatives of the species *S. pyogenes*, *S. dysgalactiae*, and *S. canis* based on HC245 clusters

Supplementary Data 15: KEGG modules abundances in all analyzed genomes

Supplementary Data 16: Kruskal-Wallis test results for KEGG module abundance across three *Streptococcus* species.
