# Supplementary material for: An ancient genome of Streptococcus pyogenes from a pre-Columbian Bolivian mummy
Source: Nat Commun. 2026 Apr 13;17:4516. doi: 10.1038/s41467-026-71603-9 (PMC13190766; doi:10.1038/s41467-026-71603-9)
Supplement: Supplementary file 4 — Reporting Summary [file 41467_2026_71603_MOESM4_ESM.pdf]

nature portfolio

Corresponding author(s): Guido Valverde
Frank Maixner

Last updated by author(s): Mar 11, 2026

Reporting Summary

Nature Portfolio wishes to improve the reproducibility of the work that we publish. This form provides structure for consistency and transparency in reporting. For further information on Nature Portfolio policies, see our Editorial Policies and the Editorial Policy Checklist.

Statistics

For all statistical analyses, confirm that the following items are present in the figure legend, table legend, main text, or Methods section.

n/a	Confirmed	
☐	☒	The exact sample size (*n*) for each experimental group/condition, given as a discrete number and unit of measurement
☐	☒	A statement on whether measurements were taken from distinct samples or whether the same sample was measured repeatedly
☐	☒	The statistical test(s) used AND whether they are one- or two-sided<br>*Only common tests should be described solely by name; describe more complex techniques in the Methods section.*
☒	☐	A description of all covariates tested
☒	☐	A description of any assumptions or corrections, such as tests of normality and adjustment for multiple comparisons
☐	☒	A full description of the statistical parameters including central tendency (e.g. means) or other basic estimates (e.g. regression coefficient) AND variation (e.g. standard deviation) or associated estimates of uncertainty (e.g. confidence intervals)
☐	☒	For null hypothesis testing, the test statistic (e.g. *F*, *t*, *r*) with confidence intervals, effect sizes, degrees of freedom and *P* value noted<br>*Give P values as exact values whenever suitable.*
☐	☒	For Bayesian analysis, information on the choice of priors and Markov chain Monte Carlo settings
☒	☐	For hierarchical and complex designs, identification of the appropriate level for tests and full reporting of outcomes
☒	☐	Estimates of effect sizes (e.g. Cohen's *d*, Pearson's *r*), indicating how they were calculated

Our web collection on statistics for biologists contains articles on many of the points above.

Software and code

Policy information about availability of computer code

Data collection	Data produced in this study:<br><br>-Shotgun genomic data for a pre-Columbian individual (ID:2730) from Bolivia was obtained.<br>-Paired-end genomic libraries of DNA extract was sequenced in an external company (Macrogen) by Illumina HiSeq-X system.<br>-De-novo assembly was performed after shotgun sequencing.<br>-Radiocarbon dating (14C) of individual ID:2730 was performed at AMS Radiocarbon Lab, Institute of Energy and the Environment, Penn State University, USA
Data analysis	Software used for data analyses:<br><br>Fastp (v.0.23.1)<br>BWA (v.0.7.17)<br>SAMtools (v.1.16.1)<br>Qualimap (v.2.2.2d)<br>MapDamage 2 (v.2.2.1)<br>Schmutzi (v.1.5.6)<br>Haplogrep3 (v.2.4.0)<br>Oxcal computer program (v.4.4.4)<br>MetaPhlAn 4 (v4.0.6)<br>AMDirT (v.1.4.5)

AMDir Ancient Metagenomics Directory)
MEGAHIT (v.1.2.9)
metaSPAdes (v.3.15.5)
MetaBAT2 (v.2.12.1)
MaxBin2 (v.2.2.7)
CONCOCT2 (v.1.1.0)
DAS Tool (v.1.1.6)
CheckM2 (v.1.0.2)
GTDBtk (v.2.1.1)
Kraken2 (v.2.17.1)
assembler Flye (v.2.9.3)
prokka (1.14.6)
BLASTp (v.2.12.0)
EnteroBase (https://enterobase.warwick.ac.uk/)
MLST (v2.23.0) (multi-locus sequence typing)
Enterobase database
PubMLST database
emmtyper
ABRicate (v1.0.0)
Virulence Factor Database (VFDB)
Comprehensive Antibiotic Resistance Database (CARD)
Panaroo (v.1.5.2)
MAFFT (v.7.525)
BEAST.X.V1.10.5
BEAGLE library
iTOL (v.7)

PHASTEST
geNomad (v1.7.4)
VIBRANT (v1.2.1)
PhageBoost (v0.1.3)
CheckV (v1.0.3)
Pharokka (v1.7.5)
-g prodigal-gv
tRNAscan-SE 2.0
Aragorn
CRT
PHROGs
VFDB
CARD
MMseqs2
PyHMMER
INPHARED
MASH
PHOLD (v0.2.0)
Foldseek
ProstT5
Phynteny (v0.1.12)
geNomad v1.7.4
Clinker (v1.3.2)
KofamScan (v1.3.0)

tidyverse (v2.0.0)
pheatmap (v1.0.13)

For manuscripts utilizing custom algorithms or software that are central to the research but not yet described in published literature, software must be made available to editors and reviewers. We strongly encourage code deposition in a community repository (e.g. GitHub). See the Nature Portfolio guidelines for submitting code & software for further information.

Data

Policy information about availability of data

All manuscripts must include a data availability statement. This statement should provide the following information, where applicable:

- Accession codes, unique identifiers, or web links for publicly available datasets
- A description of any restrictions on data availability
- For clinical datasets or third party data, please ensure that the statement adheres to our policy

Sequencing data and the assembled genome are available at the European Nucleotide Archive (ENA) under ENA: PRJEB91735. The sequencing reads are available at the Sequence Read Archive under accession ERR15308372 and the assembly under the accession number GCA_982145515.1.

Research involving human participants, their data, or biological material

Policy information about studies with human participants or human data. See also policy information about sex, gender (identity/presentation), and sexual orientation and race, ethnicity and racism.

Reporting on sex and gender	We assign the biological sex (XY) of the ancient individual (see Methods). Then, our genetic findings do not provide any information on the gender identity of these individual.
Reporting on race, ethnicity, or other socially relevant groupings	N/A
Population characteristics	N/A
Recruitment	N/A
Ethics oversight	N/A

Note that full information on the approval of the study protocol must also be provided in the manuscript.

Field-specific reporting

Please select the one below that is the best fit for your research. If you are not sure, read the appropriate sections before making your selection.

☐ Life sciences ☐ Behavioural & social sciences ☒ Ecological, evolutionary & environmental sciences

For a reference copy of the document with all sections, see nature.com/documents/nr-reporting-summary-flat.pdf

Ecological, evolutionary & environmental sciences study design

All studies must disclose on these points even when the disclosure is negative.

Study description	Metagenomic analysis of archaeological pre-Columbian human remains from Bolivia
Research sample	Archaeological human dental samples from a mummified Bolivian individual
Sampling strategy	We collected a tooth sample from a partially mummified head belonging to the anthropological collection of the National Museum of Archaeology in La Paz, Boivia (Sample ID: 2730 / Museum ID: MUNARQ 2-87)
Data collection	The sample was collected by Guido Valverde during a 2018 sampling campaign at the National Museum of Archaeology. Standard protocols were followed. A tooth sample from individual ID:2730 was secured in an individually labeled ziplock bag
Timing and spatial scale	Sample was dated to 1283–1383 cal AD
Data exclusions	No data was excluded after shotgun genomic sequencing. Data was used for downstream analyses.
Reproducibility	Analyses are described in the Method section and in the Supplementary information
Randomization	N/A
Blinding	Blinding is not applicable for ancient specimens

Did the study involve field work? ☒ Yes ☐ No

Field work, collection and transport

Field conditions	The mummified human remains analysed in this study are curated by the National Museum of Archaeology – MUNARQ in Bolivia. All necessary permits and authorizations for the study were obtained from the Bolivian Ministry of Cultures, Decolonization and Depatriarchalization (AUTORIZACIÓN MDCyT – UDAM No. 017/2018).
Location	The National Museum of Archaeology – MUNARQ in La Paz, Bolivia
Access & import/export	All necessary permits and authorizations for the study were obtained from the Bolivian Ministry of Cultures, Decolonization, and Depatriarchalization (AUTORIZACIÓN MDCyT – UDAM No. 017/2018).<br>Sample was exported from Bolivia under the AUTORIZACIÓN MDCyT – UDAM No. 017/2018 permits.<br>Eurac Research and the Bolivian Ministry of Cultures have signed a memorandum of understanding (MOU) in June, 2022 highlighting the framework of the collaboration.

Disturbance	To preserve the integrity of the remains, a minimal sampling approach was used to obtain a tooth sample (molar) from individual ID:2730. The material was subsequently processed for DNA extraction and radiocarbon (14C) dating.

Reporting for specific materials, systems and methods

We require information from authors about some types of materials, experimental systems and methods used in many studies. Here, indicate whether each material, system or method listed is relevant to your study. If you are not sure if a list item applies to your research, read the appropriate section before selecting a response.

Materials & experimental systems

n/a	Involved in the study
☒	☐ Antibodies
☒	☐ Eukaryotic cell lines
☐	☒ Palaeontology and archaeology
☒	☐ Animals and other organisms
☒	☐ Clinical data
☒	☐ Dual use research of concern
☒	☐ Plants

Methods

n/a	Involved in the study
☒	☐ ChIP-seq
☒	☐ Flow cytometry
☒	☐ MRI-based neuroimaging

Palaeontology and Archaeology

Specimen provenance	Sample belongs to a mummified pre-Columbian individual recovered from the Andean Altiplano in Bolivia.<br>Dental sample was collected from a mummified head curated at the National Museum of Archaeology, La Paz, Bolivia.<br>Permit Number: AUTORIZACIÓN MDCyT – UDAM No. 017/2018
Specimen deposition	The individual ID:2730 (Museum CODE: 2-87) is part of the Anthropological collection of the National Museum of Archaeology - MUNARQ in La Paz, Bolivia.<br>The individual is curated at the MUNARQ and remains availbale upon request from other researchers.
Dating methods	Radiocarbon dates were obtained from the AMS Radiocarbon Lab, Institute of Energy and the Environment, Penn State University, USA . Details on the sample selection and protocol can be found in the Supplementary Table S1

☒ Tick this box to confirm that the raw and calibrated dates are available in the paper or in Supplementary Information.

Ethics oversight	Information is provided in the section: Ethics & Inclusion statement<br>This research was conducted in accordance with ethical guidelines for the treatment of human remains<br>Permits granted by The National Museum of Archaeology - MUNARQ - Ministry of Cultures Decolonization, and Depatriarchalization from Bolivia.<br>Permit Number: AUTORIZACIÓN MDCyT – UDAM No. 017/2018

Note that full information on the approval of the study protocol must also be provided in the manuscript.

Plants

Seed stocks	N/A
Novel plant genotypes	N/A
Authentication	N/A
